# Short-term efficiency of plasma exchange in combination with immunosuppressants and/or biologics in the treatment of idiopathic inflammatory myopathy with rapidly progressive interstitial lung disease: a systematic review and meta-analysis

**DOI:** 10.1080/07853890.2024.2411605

**Published:** 2024-10-09

**Authors:** Yang Yang, Yan-Ting Yang, Rong-Xiu Huo, Dan-Li Meng, Xin-Xiang Huang, Jin-Ying Lin

**Affiliations:** Department of Rheumatology and Immunology, People’s Hospital of Guangxi Zhuang Autonomous Region, Nanning, China

**Keywords:** Plasma exchange, idiopathic inflammatory myopathy, myositis, rapidly progressive interstitial lung disease

## Abstract

**Objective:**

Rapidly progressive interstitial lung disease (RP-ILD) is a frequent and serious manifestation of idiopathic inflammatory myopathy (IIM) associated with poor outcomes. Plasma exchange (PE) can quickly remove pathogenic substances from the blood. Therefore, PE may be efficacious in IIM patients who have elevated levels of autoantibodies, cytokines and chemokines, fighting for time for immunosuppressive therapy. However, the value of adding PE to immunosuppressants remains unclear. The purpose of this study was to determine the short-term outcomes, including the survival rate at 6 months and change of the laboratory data, of PE in combination with immunosuppressants and/or biologics in the treatment of IIM-RP-ILD.

**Methods:**

We searched PubMed, Embase and Cochrane Library to find reports of interest published from inception to March 4, 2024. STATA 15.1 was used for data analysis. A fixed or random-effects model with inverse-variance weighting was used to estimate the pooled risk ratio (RR) and 95% confidence interval (CI).

**Results:**

Two hundred and thirty studies were identified. Eleven studies, including five retrospective cohort studies, four case-control studies and two case series, were included. PE was performed on 114 patients. The survival rate at 6 months was 80% (95%CI = 64%–92%), with moderate heterogeneity (*I*^2^=63.45%, *p* < 0.05). Moreover, the 6-month survival rate was significantly better in the PE group than in the non-PE group (RR, 1.34; 95% CI = 1.05–1.71, *I*^2^=30.7%; *p* = 0.194). ILD-related serum markers, including ferritin, KL-6 and anti-MDA-5 antibody titres, were significantly suppressed by a series of PE treatments (*p* < 0.05).

**Conclusion:**

The application of PE therapy plus treatment with corticosteroids, immunosuppressants and/or biologics was effective for patients with IIM-RP-ILD. PE may have additional supportive effect in intractable disease.

## Introduction

Dermatomyositis (DM), polymyositis (PM), overlap myositis, necrotizing autoimmune myositis and sporadic inclusion-body myositis, collectively termed idiopathic inflammatory myopathy (IIM), also known as myositis, are a group of rare, highly heterogeneous systemic rheumatic disorders characterized by skeletal muscle weakness and extra-muscular symptoms such as skin rash, dysphagia and interstitial lung disease (ILD). Rapidly progressive ILD (RP-ILD) is a particular and frequent subset of IIM-related ILD, especially in Asian patients, characterized by the deterioration of radiological interstitial alterations, dyspnoea and hypoxaemia over several weeks or a few months [1, 2]. RP-ILD has an extremely high mortality rate, which is perhaps as high as 59.5% at 3 months [3], and is the leading cause of death in anti-melanoma differentiation-associated gene 5 (MDA5) antibody-positive DM. In a large Chinese cohort study of patients with MDA5-DM, of the 121 patients who died during the observation period, 115 (95.0%) died due to respiratory failure. Of the non-survivors, 74.4% died within 6 months of disease onset [4]. Patients, notably those in the RP-ILD group, who survived the first year usually showed a significant improvement in serological markers and pulmonary function during long-term follow-up [5].

Combined immunosuppressive therapy using high-dose glucocorticoids (GC), calcineurin inhibitors and intravenous cyclophosphamide (CYC) was found to be useful in DM patients with RP-ILD. The prognosis of RP-ILD patients treated with immunosuppressive combination regimen was significantly better than that of historical controls treated with conventional step-up therapy (6-month survival rates were 75.0% and 28.6%, respectively, *p* = 0.038) [6]. Recently, the efficacy of biologics and Janus kinase inhibitors (JAKi), such as rituximab and tofacitinib (TOF) for DM-RP-ILD refractory to immunosuppressive treatment has also been reported [7, 8]. However, even when immunosuppressants and/or biologics have been used, there were still some patients who could not be saved. Failure of these treatments appears to be partly related to the rapid progression of alveolar injury before achieving ­sufficient immunosuppression [9]. In addition, opportunistic infections associated with intensive immunosuppressive therapy are concerning. Thus, additional treatment strategies should be considered in such refractory cases.

The origin of IIM-RP-ILD remains unclear, but studies have shown that the levels of many serum biomarkers, such as ferritin, Krebs von den Lungen-6 (KL-6), anti-MDA5 antibodies, IL-6 and IL-8, are high in the ILD subset [10]. Moreover, these markers were found to be related to disease activity and prognosis of IIM-RP-ILD [11–14]. Plasma exchange (PE) is an extracorporeal blood purification therapeutic procedure in which a patient’s plasma is removed from whole blood and replaced with human plasma and/or albumin solution [15]. PE can rapidly remove pathogenic molecules from the blood, such as autoantibodies, immune complexes and inflammatory cytokines. Therefore, PE may be efficacious in patients with IIM who have elevated autoantibodies, cytokines and chemokines, fighting for time for other pharmacological therapies. However, it is still unknown whether PE is effective for IIM-RP-ILD. New researches with conflicting results continue to emerge [16, 17]. Here, we performed a systematic review and meta-analysis of literature aimed at evaluating the short-term effect of PE in combination with immunosuppressants and/or biologics on the survival rate at 6 months and other clinical characteristics of patients with IIM-RP-ILD.

## Methods

This systematic review and meta-analysis were elaborated according to the guidelines of preferred reporting items for systematic reviews and meta-analysis (PRISMA). The review protocol was registered in the Prospero database (CRD42024530764).

### Search strategy

Two authors independently searched published articles indexed in databases PubMed, Embase.com and the Cochrane Library from their inception to March 4, 2024, using the MeSH terms ‘myositis/idiopathic inflammatory myopathy’, ‘lung diseases, interstitial’ and ‘plasma exchange/plasmapheresis’. The search terms included indexed terms from Medical Subject Headings (MeSH) in PubMed, Emtree in Embase.com, as well as free-text terms. A manual search of the references of selected studies was also conducted.

### Inclusion and exclusion criteria

The inclusion criteria were as follows: (1) adult patients were diagnosed with IIM-RP-ILD and PE treatments were reported; (2) studies reporting outcomes of interest; (3) randomized controlled trials, case-control, cohort studies and case series or case reports with more than five participants were included in this review; and articles were published in English or Chinese. Exclusion criteria included patients diagnosed with juvenile IIM, conference abstracts and reviews. When a cohort of patients was reported in more than one study, the paper having larger sample size was included.

### Data extraction and quality assessment

Two reviewers independently extracted data from each study using a standardized extraction sheet. The following information were collected from each study: last name of the first author, title, publication year, country where the study was conducted, study design, IIM types, anti-MDA5 antibody status, number of RP-ILD patients receiving PE, proportion of females and the mean age of the PE group, PE therapy regimen and additional biologic disease-modifying antirheumatic drugs (bDMARDs) and/or JAKi usage in the PE group. Conflicts were resolved by discussion and consensus.

Two investigators used the Newcastle-Ottawa quality assessment scale (NOS) [18] and the Joanna Briggs Institute (JBI) critical appraisal tools [19] to assess the quality of the included trials independently according to the study types. The former was used for cohort and case-control studies, whereas the latter was used for case series and intervention studies. The maximum attainable NOS score was 9, and the studies were categorized as good (7–9), moderate (3–6), or low quality (0–2) in our paper. The JBI critical appraisal checklist for case series includes ten questions. Possible answers were ‘yes’, ‘unclear’, ‘no’ or ‘not applicable’ [19]. As recommended by the JBI reviewer’s manual [20], the study was classified as low, moderate, or high quality, when the ‘yes’ scores were 0%–49%, 50%–69% and 70%–100%, respectively.

### Statistical analysis

Adjusted point estimates and standard errors were extracted from individual studies and combined using the generic inverse-variance method of DerSimonian and Laird which assigned the study’s weight based on its standard error [21]. Statistical heterogeneity was determined using the *I*^2^ statistic. It was regarded as high, moderate, low, or insignificant heterogeneity when *I*^2^ test statistics results were 76%–100%, 51%–75%, 26%–50% and 0%–25%, respectively [22]. The random-effects model was applied when *I*^2^ >50%, otherwise, we used a fixed-effects model. If necessary, the possible causes of heterogeneity were explored through subgroup analysis. Publication bias was assessed using Begg’s test. Besides, sensitivity analysis was used to estimate whether the results were stable and reliable. All calculations and graphs were performed using STATA 15.1 (StataCorp.).

## Results

### Review of the literature and included studies

**Figure 1.**
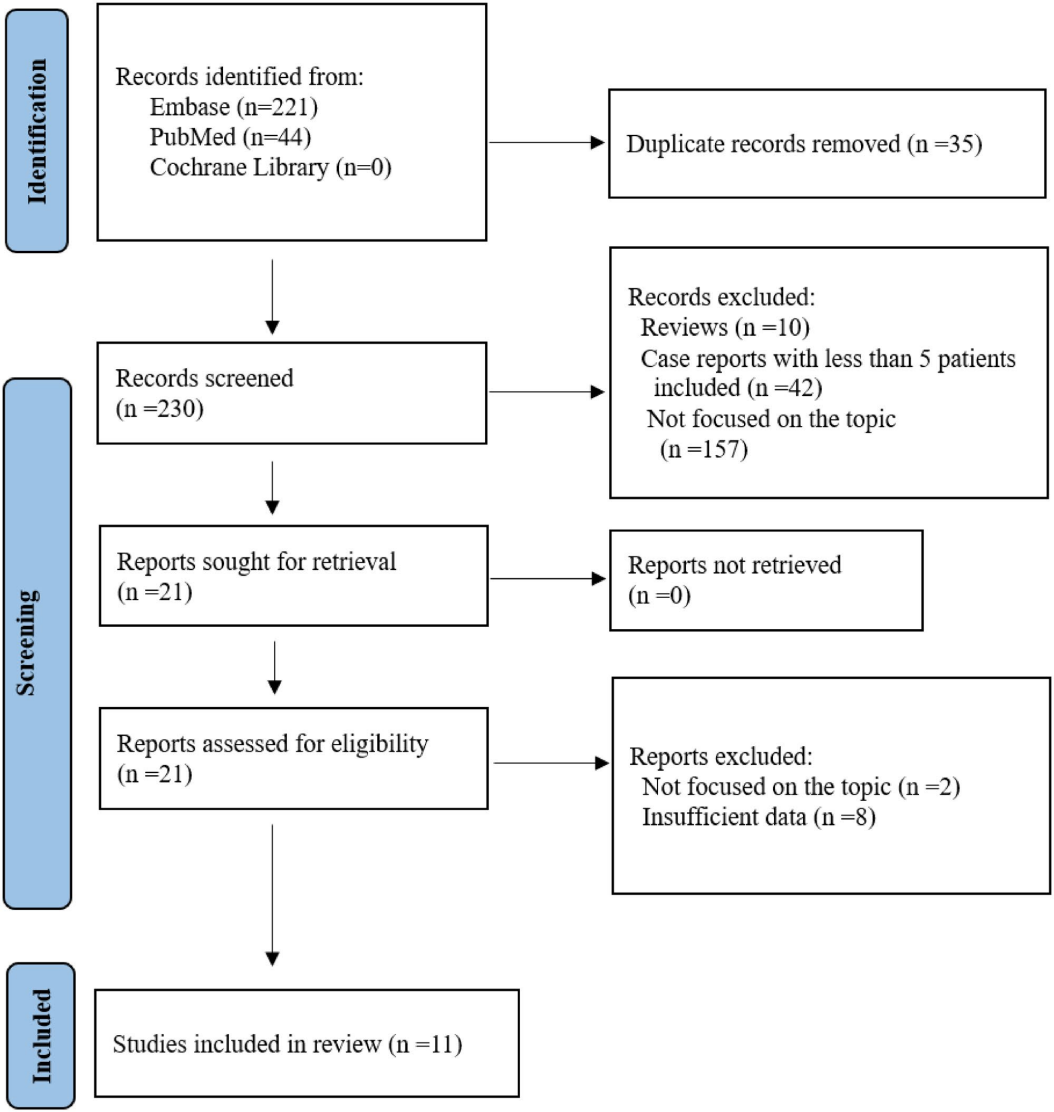
Flow diagram of manuscript selection.

Up to March 4, 2024, the literature search identified 265 articles (44 articles from PubMed and 221 articles from Embase). After reading the title, abstract and full text, we obtained 11 eligible studies, including 5 retrospective cohorts, 4 case-control studies and 2 case series [16, 17, 23–31]. The process of study identification and exclusion are summarized in Figure 1.

The characteristics of the included studies and participants are shown in Table 1. 72.7% (8/11) of the studies were conducted in Asian countries. PE was performed on 114 patients. The mean age of the PE subjects was 51.8–65.0 years. The female-to-male ratio was approximately 1.6:1 across all articles. Anti-MDA5 antibodies tested positive for most patients. There were discrepancies in the PE treatment regimens across different studies. Additional bDMARDs used for patients in the PE group included rituximab (*n* = 21), abatacept (*n* = 2), tocilizumab (*n* = 1) and infliximab (*n* = 1). Besides, twenty-one patients were treated with a combination of JAKi.

**Table 1.** Main characteristics of studies included in the meta-analysis.

Author	Year	Country	PE group, n	Mean age (y)	Female (%)	IIM types, n	Anti-MDA5 positive	PE therapy regimen		Additional bDMARDs and/or JAKi usage	Quality assessment
								Time interval	Times		
Vuillard et al. [30]	2018	France	8	60.0	51.1	DM: 7; ASS: 1	NA	NA	NA	NA	Good
Ning et al. [26]	2019	China	18	54.5	66.7	DM: 5; CADM: 11; PM: 2	NA	Every day for 3 days, and every other day after that	5 (4–24)	N	Good
Abe et al. [23]	2020	Japan	6	62.0	50.0	NA	Y	NA	16 (1–23)	N	Good
Shirakashi et al. [16]	2020	Japan	8	56.0	50.0	DM: 5; CADM: 3	Y	One to three times per week	11.4 ± 3.4	N	Good
Komai et al. [25]	2021	Japan	11	57.0	72.7	DM/CADM	Y	One to three times per week	17	RTX: 3; TOF: 4; ABA: 2	Good
Saito et al. [27]	2021	Japan	6	56.0	66.6	CADM	Y	Every other day	9.5 (3–14)	RTX: 2	Good
Bay et al. [17]	2022	France	25	52.0	64.0	DM	Y	NA	5 (3–6)	RTX: 5; JAKi: 8; IFX: 1	Good
Shirai et al. [29]	2023	Japan	8	51.8	62.5	DM	Y	Three days per week	NA	RTX: 8; TOF: 8	Mod
Eggleston et al. [24]	2023	Minnesota	9	55.0	56.0	NA	Y*	Every other day/daily	5	RTX: 3	Good
Watanabe et al. [31]	2023	Japan	6	65.7	50.0	CADM	Y	Two to three times a week	16.3 ± 5.0	TOF: 1 TCZ: 1	Mod
Sasaki et al. [28]	2024	Japan	9	53.3	88.9	DM: 2; CADM: 7	Y	Three times within a week	9 (4.5)	N	Good

PE, plasma exchange; IIM, idiopathic inflammatory myopathy; DM, dermatomyositis; CADM, clinically amyopathic DM; PM, polymyositis; ASS, anti-synthetase syndrome; Anti-MDA5, anti-melanoma differentiation-associated gene 5; Y, yes; N, no; NA, not applicable; bDMARDs, biologic disease-modifying antirheumatic drugs; TOF, tofacitinib; RTX, rituximab; ABA, abatacept; JAKi, Janus kinase inhibitors; IFX, infliximab; TCZ, tocilizumab; *Anti-MDA5, detected in 33% of patients.

### Survival rate at 6-month

**Figure 2.**
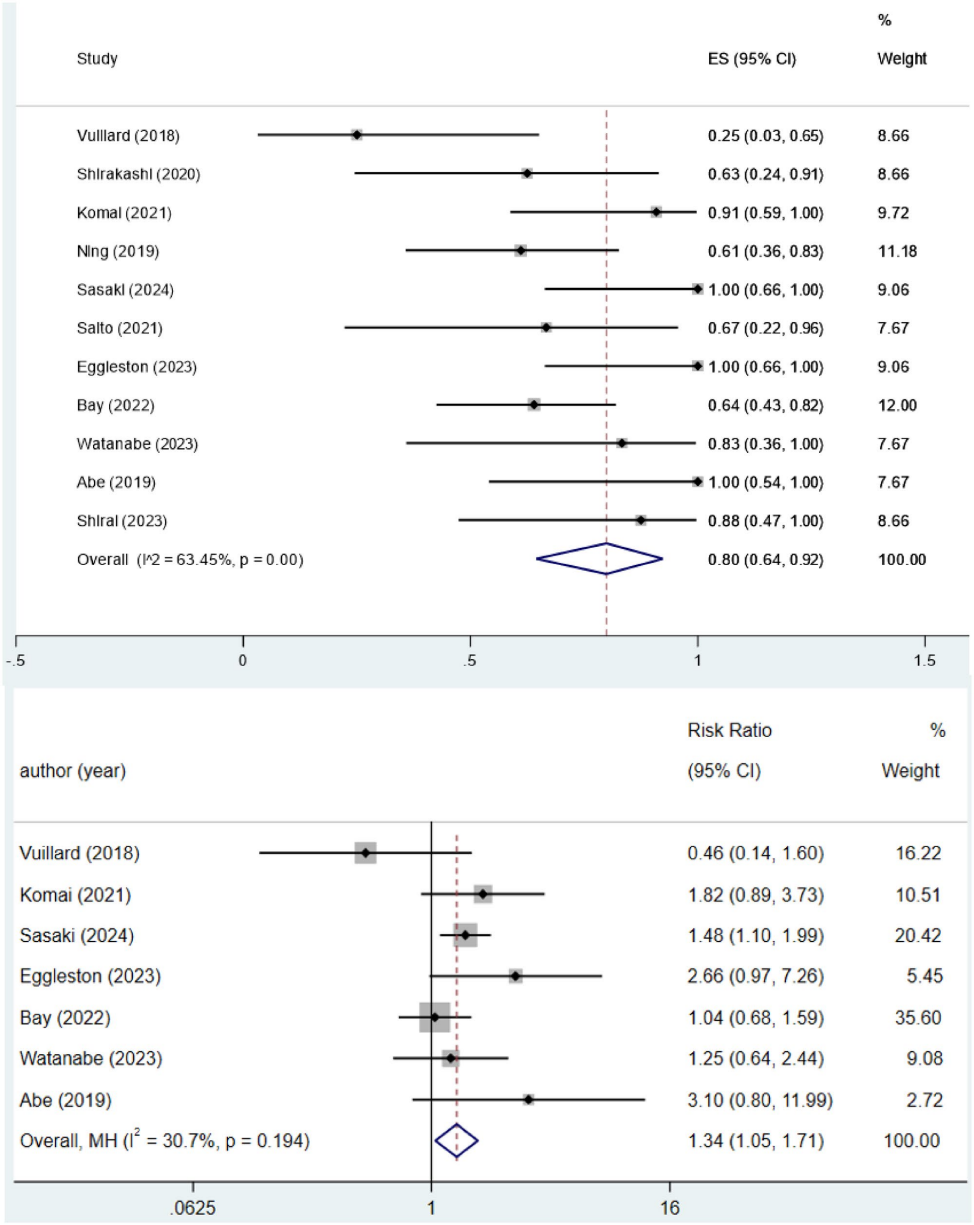
(A) Survival rate at 6 months of rapid progressive interstitial lung disease in idiopathic inflammatory myopathy patients treated with plasma exchange. (B) 6-month survival rate of the plasma exchange group was higher than that of the non-plasma exchange group.

The survival rate at 6-month was 80% (95%CI = 64%–92%), with moderate heterogeneity (*I*^2^=63.45%, *p* < 0.05; Figure 2A). The 6-month survival rate of the PE group was higher than that of the non-PE group (RR, 1.34; 95% CI = 1.05–1.71, *I*^2^=30.7%; *p* = 0.194; Figure 2B).

### Comparison of laboratory data before and after PE

Five studies evaluated changes in laboratory biomarkers in patients before and after PE [16, 25, 27, 28, 31]. The titres of ILD-related serum markers, including ferritin, KL-6 and anti-MDA5 antibody, were significantly suppressed by a series of PE treatments (Table 2).

**Table 2.** Clinical parameter changes before and after PE in PE-treated IIM-RP-ILD patients.

Author	Clinical makers	Before PE treatment	After PE treatment	*p*-value
	Ferritin (ng/ml)	1620.0 (931.8–4842.6)	167.0 (57.8–297.1)	<0.05
Shirakashi et al. [16]	KL-6 (U/ml)	1535.0 (942.3–1942.5)	876.0 (312.5–1654.5)	<0.05
	Anti-MDA5 antibodies (U/ml)	159.0 (144.1–215.2)	36.8 (18.8–56.6)	<0.05
	Ferritin (ng/ml)	NA	NA	<0.05
Komai et al. [25]	KL-6 (U/ml)	NA	NA	<0.05
	Anti-MDA5 antibodies (index)	NA	NA	<0.05
	Ferritin (ng/ml)	2145	21	0.004
Sasaki et al. [28]	KL-6 (U/ml)	1089	595	0.008
	Anti-MDA5 antibodies (U/ml)	>32	<32	NA
	Ferritin (ng/ml)	830–2867	NA	*NA
Saito et al. [27]	KL-6 (U/ml)	351–1689	NA	*NA
	Anti-MDA5 antibodies (U/ml)	150–250	<100	*NA
Watanabe et al. [31]	Ferritin (ng/ml)	2010.2 ± 557.0	979.3 ± 436.8	0.016

PE, plasma exchange; IIM, idiopathic inflammatory myopathy; RP-ILD, rapidly progressive interstitial lung disease; KL-6, Krebs von den Lungen-6; NA, not applicable; *The author declared that the levels of KL-6, ferritin and anti-MDA5 antibodies decreased after PE.

### Subgroup analysis

To further explore the moderate level of heterogeneity seen in our results, we conducted a subgroup analysis based on the study design (cohort *vs*. non-cohort studies), region (Asian *vs*. not-Asian countries) and use of bDMARDs and/or JAKi (Yes *vs*. No). According to the region, the 6-month survival rate of the PE group was lower in not-Asian countries (69%) compared to Asian countries (84%). In the subgroup analysis based on study type, the RP-ILD survival rate was greater in the cohort (85%) than in the non-cohort studies (75%). The survival rate at 6 months in the PE plus bDMARDs and/or JAKi therapy group did not seem to be better than that of the group without bDMARDs and/or JAKi treatment (83% *vs.*85%); however, this finding should be interpreted with great caution and must be confirmed to further prospective studies, as the studies included in this paper, those that administered bDMARDs and/or JAKi to patients indicated only a subset of patients who received the drugs mentioned above. Table 3 shows the results of the subgroup analysis.

**Table 3.** Results of subgroup analysis according to regions, study designs and use of bDMARDs and/or JAKi.

Subgroups	N	Survival rate at 6-month	95% CI	*P* within^a^	*I* ^2^	*P* between^b^
Overall	11	80%	64–92	NA	63.45%	<0.05
Regions						
Asia	8	84%	69–95	0.11	40.07%	0.511
Non-Asia	3	69%	25–99	NA	NA	
Study designs						
Cohort	5	85%	64–99	0.04	61.16%	0.482
Non-cohort	6	75%	50–94	0.01	69.64%	
Use of bDMARDs and/or JAKi						
Yes	6	83%	68–95	0.14	39.58%	0.913
No	4	85%	56–100	0.02	70.53%	

bDMARDs, biologic disease-modifying antirheumatic drugs; JAKi, Janus kinase inhibitors; NA, not applicable; a, *P* for heterogeneity within subgroups; b, *P* for heterogeneity between subgroups.

### Risk of publication bias and sensitivity analysis

**Figure 3.**
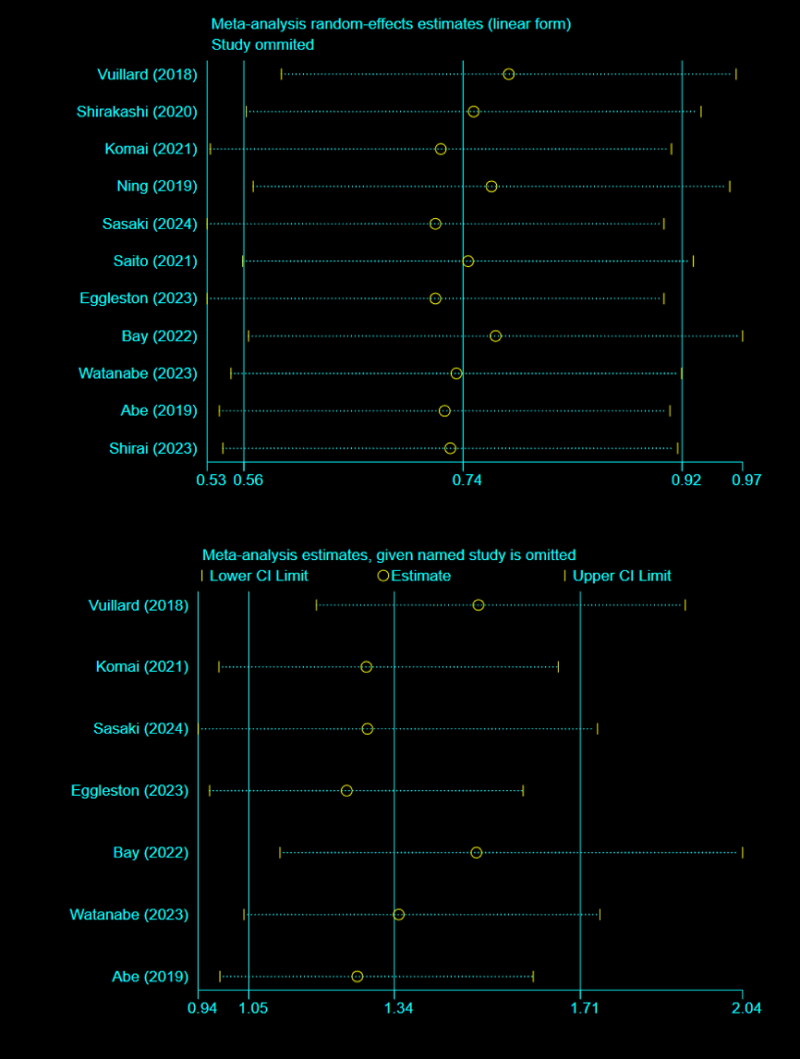
Sensitivity analysis.

There was no evidence of publication bias detected by Begg’s test (*p* > 0.05). Additionally, the sensitivity test demonstrated the results were robust and reliable (Figure 3).

## Discussion

To the best of our knowledge, this study is the first meta-analysis to compare the difference in short-term survival between PE- and non-PE-treated patients with myositis-related RP-ILD. In our review, 114 IIM-RP-ILD subjects undergoing PE were included, with a 6-month survival rate of 80% (95%CI = 64%–92%), and the survival rate of patients in the PE group was much better than that in the non-PE group (RR: 1.34, 95% CI = 1.05–1.71, *I*^2^=30.7%, *p* = 0.194). A previous meta-analysis conducted by Wang et al. [32] displayed the 6-month survival rate of DM/PM-RP-ILD patients treated with PE was only 55.8% (95% CI = 26.4%–85.2%), but they just included four studies with 45 patients. The difference in sample size may be one reason for the discrepancy in the results. Furthermore, the main purpose of the review by Wang et al. was not to evaluate the impact of PE on the prognosis of patients with IIM-RP-ILD.

IIM-related RP-ILD is a disease requiring early intervention due to poor overall prognosis. Treatment delays, even for a few weeks, can greatly affect the outcome in such patients, indicating the importance of initiating valid and aggressive treatment as soon as possible in these persons. Standard treatment strategies for IIM-RP-ILD have not yet been established, but some experts have suggested algorithms that suggested early and intensive immunosuppressive therapy [33–35]. Initiating high-dose steroids in conjunction with immunosuppressive drugs such as tacrolimus, CYC and mycophenolate mofetil is the cornerstone of therapy. However, in clinical practice, there are still some patients who do not respond well to the combination regimen mentioned above and die quickly because their rapid disease progression overcomes the therapeutic effects of early stage of the disease.

The benefit of PE in GC-resistant PM/DM was first reported by Dau et al. in 1981 [36]. All patients received immunosuppressive agents in association with PE. The benefit of this combined therapy was noted in 23 of the 26 patients. Since then, an increasing number of scholars have conducted related research. Recently, Sasaki et al. [28] reported that the PE therapy, in addition to intensive immunosuppressive treatment, was effective in patients with anti-MDA5-positive DM and severe and refractory RP-ILD. They discovered all nine patients in the PE group had improved respiratory symptoms and were alive, whereas 12 of 31 patients in the non-PE group died (100% *vs.* 61%, *p* = 0.038). In another study, PE was performed in 8 DM patients who still had progressed to RP-ILD after combined immunosuppressive therapy, of which 5 survived; however, the 5 RP-ILD subjects who did not receive PE died (62.5% *vs.* 0%, *p* = 0.04). All deaths occurred within 6 months after treatment. The additional treatments used for those RP-ILD patients without PE were biologics [anti-TNF-α (*n* = 1) or anti-IL-6 (*n* = 2)], intravenous immunoglobulin (*n* = 1), or weekly intravenous CYC (*n* = 1) [16]. In 2019, the use of TOF was initiated in IIM participants. Lately, Shirai et al. [29]displayed intensive induction therapy consisting of combinations of triple therapy (GC, CYC and tacrolimus) with higher doses of GC, PE, rituximab and TOF significantly improved the survival of patients with severe anti-MDA5-RP-ILD. Dead patients were characterized by older age, elevated C-reactive protein level and high serum ferritin level. The authors argued that intensive induction treatment would be an option for patients aged 30–60 with multiple poor prognostic factors, particularly those with very high serum ferritin levels, although more research is needed to confirm this. Several adverse events were observed in the group, the most common of which was reactivation of cytomegalovirus (75%). Our report and the original studies included from Japan suggested the efficacy of PE in IIM-RP-ILD patients. However, PE did not seem to provide advantage to MDA5-DM-RP-ILD patients within the French cohort [17, 30]. The inclusion of more severe patients in their study may be partly to blame. According to Bay et al. [17], 40 (78%) patients in their research required intensive care unit admission for acute respiratory failure. Mechanical ventilation and extracorporeal life support were needed in 63% and 31% cases, respectively. However, many cases reported from Japan had low oxygen requirements and few subjects admitted to intensive care unit and received mechanical ventilation. This may be attributed to the fact that anti-MDA5 antibodies can be measured in general clinics, and access to the university hospitals is easier in Japan. The difference in sample sizes may represent an additional potential factor contributing to the observed discrepancies in the results. The samples in these Japanese studies were small. In addition, the number of PE in the cohort by Bay et al. was lower than in other studies, such as the trial by Shirakashi et al. [16] (5[3–6] *vs.*11.4 ± 3.4). The clinical situation in which PE is used is inconsistent across different articles. Hence, the results need to be interpreted with caution and must be confirmed to further prospective studies.

The benefit of PE in myositis-RP-ILD may be related to autoantibodies (especially anti-MDA5), cytokines and/or chemokine direct/indirect pathogenicity, which remains unclear. Previous studies indicated that MDA5 autoantibody titres correlated with the gravity, activity and risk of relapse of DM-related RP-ILD and could lead to type I interferon (IFN) response and inflammatory cytokine activation [37]. CX3CL1, a type I IFN-related chemokine, is associated with anti-MDA5 antibody titre [38]. Interestingly, CX3CL1 expression can be induced by type I IFN in pulmonary vascular endothelial cells [39] and is thought to drive the migration of CX3CR1^-^positive nonclassical monocytes to the lungs of patients with ILD, thereby perpetuating the local fibrotic process [40]. The expression of type I IFN-related molecules correlates with ILD progression and/or patient prognosis [41], suggesting that type I IFN may play a role in lung involvement in MDA5-DM patients [42]. Elevated levels of various cytokines/chemokines have been reported to be related to ILD disease activity associated with IIM. IL-4, IL-6, IL-8, IL-10 and TNF-α levels increased in ILD with IIM [10]. IL-6, IL-8, IL-10 and serum ferritin levels were significantly higher in PM/DM patients with RP-ILD compared with non-or chronic-ILD [10, 14]. In a separate study, IL-15 concentrations in both the serum and bronchoalveolar lavage fluid were notably higher in patients with RP-ILD than in those without RP-ILD. Furthermore, levels of many cytokines/chemokines were significantly higher in intractable anti-MDA5-positive ILD cases than in those who responded well to immunosuppressive therapy [38, 43]. Hence, the association of macrophage/monocyte activation markers with disease severity and patient prognosis suggests a role for macrophage/monocyte activation in the pathogenesis of IIM-RP-ILD, especially in those with anti-MDA5 antibodies [44]. The studies included in our review showed a significant increase in anti-MDA5 antibody titres and various cytokines or proteins from monocytes/macrophages in IIM-RP-ILD before PE treatment. In this context, a cytokine storm triggered by monocyte/macrophage overactivation appears to be present in the background of serious cases of anti-MDA5-positive IIM-RP-ILD, and removal of these substances is an important therapeutic strategy.

It is necessary to standardize the PE protocol in the future. As we have described above, the baseline characteristics of patients receiving PE, as well as the time points, number and procedures of the PE regime varied across different studies. To date, the application of PE treatment was more determined by clinical discussion considering poor prognostic factors [16, 23–25, 28, 29, 31]. Based on our analysis and the included articles mentioned earlier, we tend to believe that a subset of patients with high inflammatory biomarkers, e.g. anti-MDA5 antibodies, ferritin, IL-6, IL-8, IL-10 and IL-15, may be more likely to benefit from PE. Additionally, PE might be an alternative treatment for IIM-RP-ILD patients resistant to intensive immunosuppressive therapies [16, 23]. A recent small retrospective study from Japan reported better survival (100% *vs*. 61%, *p* = 0.038) among MDA5-DM-RP-ILD patients receiving early initiation of PE (within 2 weeks after the initial combined immunosuppressive treatment). In the research by Ning et al. [26], the application of PE within 1 week after methylprednisolone administration was evaluated in 18 patients admitted to the intensive care unit due to exacerbation of ILD after failure of intensive treatment; unfortunately, 7 patients (38.9%) still died of respiratory failure. For patients requiring ministration of the intensive care unit, mechanical ventilation and extracorporeal life support, the therapeutic effect of PE seems to be limited. Currently, it is challenging to delineate ‘when’ and ‘how (how many times and how often)’ PE should be administered in patients with myositis-related RP-ILD, but our impression from this analysis is that it should be determined individually, taking into account the extent of the patient’s inflammation, severity of pulmonary interstitial involvement, prior medication use, the burden of comorbidities and the socio-economic burden.

This study has some limitations. Firstly, the data were obtained only from retrospective studies associated with different clinical settings and other parameters; thus, the results might have been affected and could have introduced bias. Secondly, due to the limited testing power of the Begg’s test, publication bias is considered possible and the possibility of false negative results cannot be ruled out. Thirdly, most of the included studies included a limited number of patients with PE. The lack of relevant data is mainly due to the low incidence of IIM. However, our meta-analysis included all relevant studies, and to some extent, the number of patients could be considered sufficient. Finally, we were unable to further analyze the long-term survival of PE-treated IIM-RP-ILD patients due to the lack of relevant data; however, as described earlier, most deaths occurred during the early stages of the disease, and RP-ILD patients who survive the first year usually tend to stabilize afterward. Additionally, long-term PE therapy is currently relatively difficult to achieve. Hence, in our opinion, we tend to suggest that whether PE could play a role in the early stages of disease is more worthy of attention.

## Conclusion

In summary, the results of this study suggest PE is expected to be an effective adjuvant treatment for IIM-RP-ILD, particularly in resistant cases. In the future, prospective studies should be conducted to determine the effectiveness of this treatment and improve the prognosis of this condition.

## Supplementary Material

Supplementary table PRISMA 2020 checklist.docx

## Data Availability

Data are available on request from the corresponding author.
